# Development of An Oral Treatment with the PPAR-γ-Acting Cannabinoid VCE-003.2 Against the Inflammation-Driven Neuronal Deterioration in Experimental Parkinson’s Disease

**DOI:** 10.3390/molecules24152702

**Published:** 2019-07-25

**Authors:** Sonia Burgaz, Concepción García, Maria Gómez-Cañas, Eduardo Muñoz, Javier Fernández-Ruiz

**Affiliations:** 1 Instituto Universitario de Investigación en Neuroquímica, Departamento de Bioquímica y Biología Molecular, Facultad de Medicina, Universidad Complutense, 28040 Madrid, Spain; 2 Centro de Investigación Biomédica en Red de Enfermedades Neurodegenerativas (CIBERNED), 28040 Madrid, Spain; 3 Instituto Ramón y Cajal de Investigación Sanitaria (IRYCIS), 28040 Madrid, Spain; 4 Instituto Maimónides de Investigación Biomédica de Córdoba (IMIBIC), 14004 Córdoba, Spain; 5 Departamento de Biología Celular, Fisiología e Inmunología, Universidad de Córdoba, 14004 Córdoba, Spain; 6 Hospital Universitario Reina Sofía, 14004 Córdoba, Spain; 7 Vivacell Biotechnology España, 14004 Córdoba, Spain

**Keywords:** cannabinoids, VCE-003.2, oral formulation, PPAR-γ, γ receptors, inflammation, Parkinson’s disease

## Abstract

In a recent study, we described the neuroprotective properties of VCE-003.2—an aminoquinone derivative of the non-psychotropic phytocannabinoid cannabigerol (CBG)—administered intraperitoneally (i.p.) in an inflammatory model of Parkinson’s disease (PD). We also demonstrated that these properties derive from its activity on the peroxisome proliferator-activated receptor-γ, in particular at a regulatory site within this receptor type. In the present study, we wanted to further confirm this neuroprotective potential using an oral lipid formulation of VCE-003.2, developed to facilitate the clinical development of this phytocannabinoid derivative. To this end, we evaluated VCE-003.2, administered orally at two doses (10 and 20 mg/kg), to mice subjected to unilateral intrastriatal injections of lipopolysaccharide (LPS), a classic model of inflammation-driven neuronal deterioration that recapitulates characteristics of PD. The administration of VCE-003.2 to these mice showed, as expected, poor activity in the different motor tests (rotarod, computer-aided actimeter) used in experimental parkinsonism, in general due to the lack of evident changes in these behaviors by LPS lesion. However, VCE-003.2, at 20 mg/kg, was highly active in improving the changes detected in LPS-lesioned mice in the cylinder rearing test. In addition, the histopathological analysis of the basal ganglia revealed a trend towards recovery at 20 mg/kg VCE-003.2 in the loss of tyrosine hydroxylase-containing nigrostriatal neurons, as well as a complete reduction in the elevated LAMP-1 immunolabeling (reflecting autophagy impairment) caused by LPS lesion. These effects were not seen at 10 mg/kg. This was associated with a partial reduction in the intense glial reactivity provoked by LPS in the substantia nigra, in particular the astroglial reactivity labeled with glial fibrillary acidic protein. The analysis using qPCR in the striatum of proinflammatory mediators, such as tumor necrosis factor-α, interleukin-1β, inducible nitric oxide synthase, and cyclooxygenase-2, showed that the marked elevations provoked by the LPS lesion tended to be, in general, attenuated by VCE-003.2 treatment, with the greatest effects normally found with the highest dose of 20 mg/kg. In summary, our data confirm the neuroprotective potential of an oral formulation of VCE-003.2 against neuronal injury in an in vivo model of PD based on neuroinflammation, and this study opens the possibility to further the development of oral VCE-003.2 in the clinic.

## 1. Introduction

Inflammation appears to be a key etiologic factor and pathogenic event in Parkinson’s disease (PD), to the point that the risk of developing PD has been found to be lower in subjects chronically treated with nonsteroidal anti-inflammatory agents for other pathological conditions [1], whereas subjects bearing inflammatory cytokine gene polymorphisms might have increased risk [2]. This has promoted the investigation of strategies against neuroinflammation to limit neuronal deterioration in this disease [3,4,5]. Cannabinoids have been found to be highly active anti-inflammatory agents [6,7], an effect that has been normally linked to the activation of the cannabinoid receptor type-2 (CB_2_), whose benefits have been already investigated in experimental models of PD [8,9,10]. The anti-inflammatory properties of cannabinoids have recently been reinforced with the observation that different phytocannabinoids and their derivatives, as well as the endocannabinoids anandamide and 2-arachidonoylglycerol, and related signaling lipids such as palmitoylethanolamide and oleylethanolamide, can also bind and activate specific receptor types of the peroxisome proliferator-activated receptor (PPAR) family [11,12]. These nuclear receptors play an important role in some cell and tissue functions and, in the case of PPAR-γ, they have long been involved in the control of neuroinflammatory responses [13]. This also includes the case of PD for which non-cannabinoid PPAR-γ activators such as glitazones have been found to be active in experimental PD models and have recently entered clinical investigation [14], but with the risk of important side effects. Therefore, the fact that some cannabinoids are able to activate PPAR-γ [12,15,16] enables these compounds to exert anti-inflammatory effects besides their CB_2_ receptor-mediated anti-inflammatory activity and they may present a safer profile.

A series of quinone derivatives of the phytocannabinoid cannabigerol (CBG), which behave as PPAR-γ activators showing negligible affinity at the CB_1_/CB_2_ receptors unlike their phytocannabinoid template, have been recently designed, synthesized, and characterized [17,18,19]. One of them, the aminoquinone derivative of CBG, so-called VCE-003.2, was recently investigated in murine models of Huntington’s disease [19] confirming its neuroprotectant profile exerted by activating PPAR-γ and its ability to cross the blood–brain barrier after systemic administration. In a more recent study [20], these anti-inflammatory and neuroprotectant properties of VCE-003.2 were investigated in lipopolysaccharide (LPS)-lesioned mice, the experimental model of PD which better reproduces inflammation as a pathogenic event in this disease [9,10]. Our data, in this in vivo PD model [20], reveal its efficacy via intraperitoneal (i.p.) administration against the inflammation-driven nigrostriatal neuronal deterioration—a fact further confirmed in in vitro studies using cultured BV2 cells stimulated with LPS as well as experiments with cultured M-213 exposed to BV2-dependent conditioned media [20]. Furthermore, the beneficial effects of VCE-003.2 were confirmed to be mediated by the activation of PPAR-γ, by binding to a functional alternative binding site within this receptor (with regulatory functions) that would be different to the canonical binding site used by glitazones [21]. We confirmed that VCE-003.2 binds to the PPAR-γ at this different site using docking and transcriptional analyses [20]. In the present study, we wanted to further investigate this neuroprotective potential in PD using an oral lipid formulation of VCE-003.2 to facilitate the development of this phytocannabinoid derivative. Therefore, we evaluated VCE-003.2, administered orally at two doses (10 and 20 mg/kg), to mice subjected to unilateral intrastriatal injections of LPS, the same model of inflammation-driven nigrostriatal neuronal deterioration used in our previous study [20].

## 2. Results

**Figure 1:**
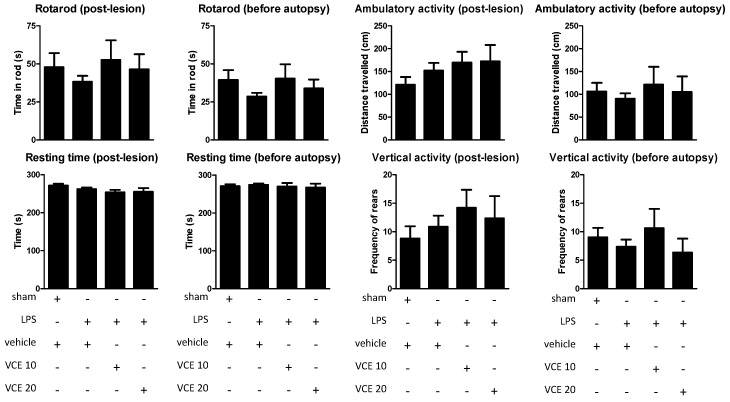
Rotarod performance, horizontal (ambulation) and vertical (rearing) activities, and resting time measured in a computer-aided actimeter in control and lipopolysaccharide (LPS)-lesioned mice orally treated for 28 days after lesion with vehicle (corn oil) or VCE-003.2 at the doses of 10 (VCE 10) or 20 (VCE 20) mg/kg. Data correspond to only one week after the onset of the treatment (post-lesion), or to only one day before the autopsy and after four weeks of treatment (before autopsy). Values are means ± SEM of more than six subjects per group. Data were assessed using the one-way analysis of variance followed by the Bonferroni test.

The oral administration of the two doses of VCE-003.2 (10 and 20 mg/kg) to LPS-lesioned mice, as expected, resulted in poor activity in two motor tests, rotarod, and computer-aided actimeter, frequently used in our laboratory and other laboratories to detect behavioral abnormalities in experimental parkinsonism (see Reference [6] for review). However, it is important to note that such behavioral abnormalities have not been seen, in general, in LPS-lesioned mice possibly because the lesion was unilateral [9,10,20], and the present study was not an exception. Thus, the rotarod performance was not altered in LPS-lesioned mice compared to control mice (post-lesion: F(3,49) = 0.508, ns; before autopsy: F(3,48) = 1.059, ns; Figure 1). Similar results were obtained using the computer-aided actimeter with the ambulatory activity (post-lesion: F(3,45) = 1.019, ns; before autopsy: F(3,49) = 0.265, ns; Figure 1), resting time (post-lesion: F(3,49) = 1.491, ns; before autopsy: F(3,49) = 0.216, ns; Figure 1), and vertical activity (post-lesion: F(3,47) = 0.553, ns; before autopsy: F(3,43) = 0.687, ns; Figure 1), as well as with other parameters, e.g., slow and fast movements, mean and maximal velocity (data not shown). Under these conditions, the possible beneficial effects of VCE-003.2 at the two doses investigated here could not be demonstrated (Figure 1), despite certain trends that could be appreciated with the dose of 10 mg/kg of VCE-003.2 (but not with 20 mg/kg). The lack of statistical significance in the above F values, degrees of freedom, and probability levels supports that these apparent trends have no biological relevance in contrast to other trends that are discussed below.

**Figure 2:**
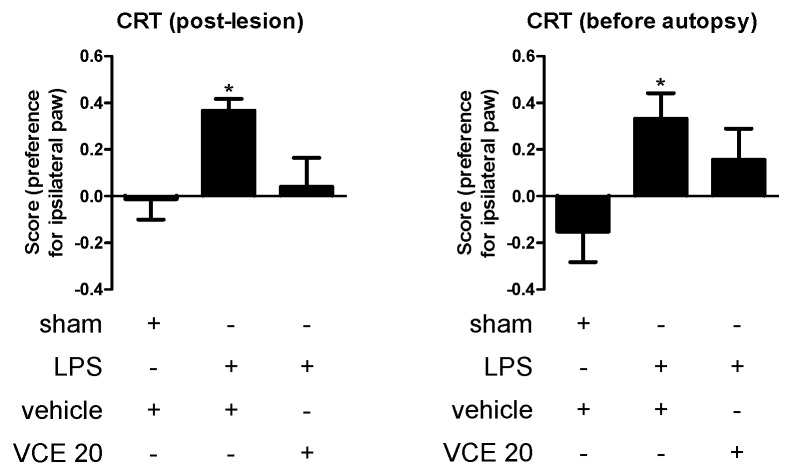
Preference for the ipsilateral paw measured in the cylinder rearing test in control and LPS-lesioned mice orally treated for 28 days after lesion with vehicle (corn oil) or VCE-003.2 at the dose of 20 mg/kg (VCE 20). Data correspond to only one week after the onset of the treatment (post-lesion), or to only one day before the autopsy and after four weeks of treatment (before autopsy). Values are means ± SEM of more than six subjects per group. Data were assessed using the one-way analysis of variance followed by the Bonferroni test (* *p* < 0.05 versus vehicle-treated control (sham) mice).

However, in a separate experiment conducted at the highest 20 mg/kg dose of VCE-003.2 after the previous data revealed no changes at the behavioral level, we were able to prove that this CBG derivative was highly active in improving the changes detected in LPS-lesioned mice in another PD-related test, the cylinder rearing test, that may be a more relevant functional endpoint when the lesion is unilateral. Our data demonstrate that LPS lesion caused a preference for the ipsilateral paw in the cylinder rearing test, which was evident at both 1 week after the onset of the treatment (F(2,30) = 4.542, *p* < 0.05; Figure 2) and directly before autopsy after 4 weeks of treatment (F(2,30) = 3.642, *p* < 0.05; Figure 2), whereas the treatment with VCE-003.2 partially corrected this alteration, in particular at 1 week after the onset of the treatment (Figure 2).

**Figure 3:**
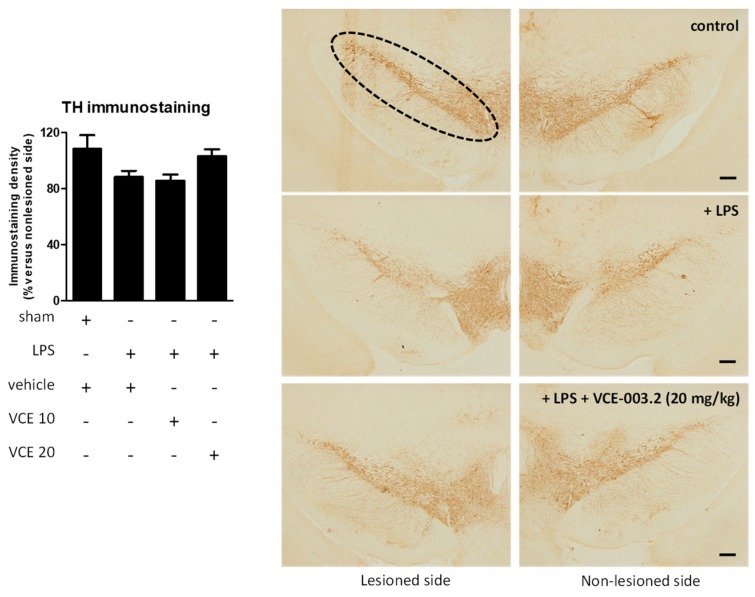
Intensity of the immunostaining for tyrosine hydroxylase (TH) measured in a selected area of the substantia nigra pars compacta of control and LPS-lesioned mice orally treated for 28 days after lesion with vehicle (corn oil) or VCE-003.2 at the doses of 10 (VCE 10) or 20 (VCE 20) mg/kg. Immunoreactivity values are measures in the lesioned side over the non-lesioned side, and correspond to means ± SEM of more than six subjects per group. Data were assessed using the one-way analysis of variance followed by the Bonferroni test. Representative immunostaining images for sham and LPS-lesioned mice treated with vehicle or VCE-003.2 at 20 mg/kg, with indication of the approximate area quantified, are shown at right (scale bar = 200 µm).

**Figure 4:**
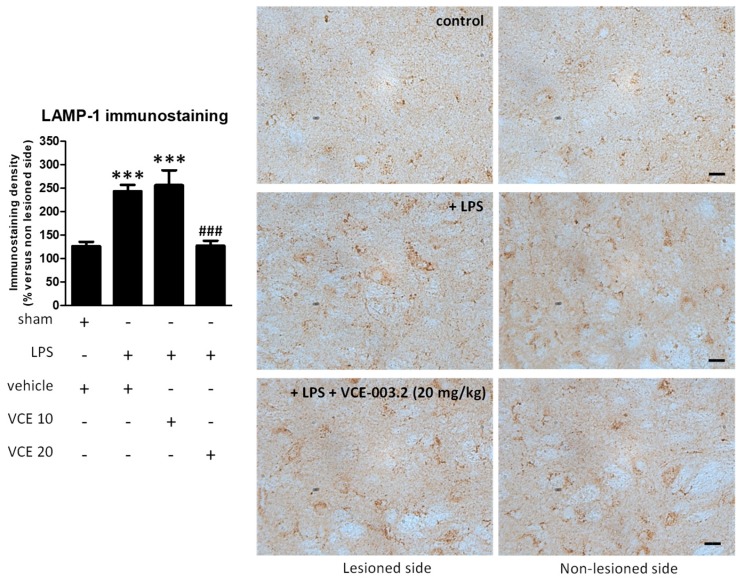
Intensity of the immunostaining for LAMP-1 measured in a selected area of the substantia nigra pars compacta of control and LPS-lesioned mice orally treated for 28 days after lesion with vehicle (corn oil) or VCE-003.2 at the doses of 10 (VCE 10) or 20 (VCE 20) mg/kg. Immunoreactivity values are measured in the lesioned side over the non-lesioned side, and correspond to means ± SEM of more than six subjects per group. Data were assessed using the one-way analysis of variance followed by the Bonferroni test (*** *p* < 0.005 versus vehicle-treated control (sham) mice; ### *p* < 0.005 versus vehicle-treated LPS-lesioned mice). Representative immunostaining images for sham and LPS-lesioned mice treated with vehicle or VCE-003.2 at 20 mg/kg are shown to the right (scale bar = 25 µm).

The histopathological analysis of the basal ganglia revealed a trend towards a recovery by VCE-003.2, at 20 mg/kg but not at 10 mg/kg, in the loss of tyrosine hydroxylase (TH)-containing nigrostriatal neurons caused by the LPS lesion (F(3,45) = 2.266, *p* = 0.095; Figure 3). We also analyzed LAMP-1, a marker of autophagy, which was elevated, reflecting autophagy dysregulation in the experimental models of PD [22,23], including LPS-lesioned mice [24], and also in biological samples of patients [25]. Our data indicate a 2-fold elevation in LAMP-1 in LPS-lesioned mice and a complete recovery after the treatment with VCE-003.2, but only at the higher dose (F(3,47) = 24.94, *p* < 0.0001; Figure 4).

**Figure 5:**
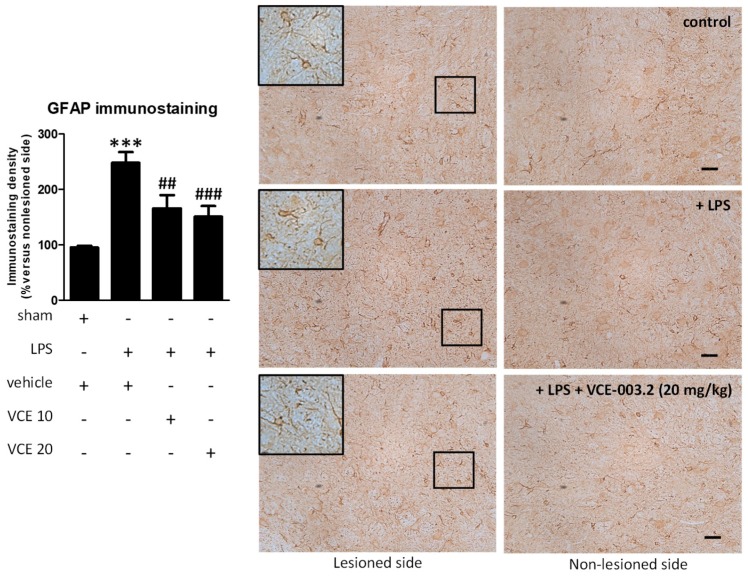
Intensity of the immunostaining for GFAP measured in a selected area of the substantia nigra pars compacta of control and LPS-lesioned mice orally treated for 28 days after lesion with vehicle (corn oil) or VCE-003.2 at the doses of 10 (VCE 10) or 20 (VCE 20) mg/kg. Immunoreactivity values are measured in the lesioned side over the non-lesioned side, and correspond to means ± SEM of more than six subjects per group. Data were assessed using the one-way analysis of variance followed by the Bonferroni test (*** *p* < 0.005 versus vehicle-treated control (sham) mice; ## *p* < 0.01, ### *p* < 0.005 versus vehicle-treated LPS-lesioned mice). Representative immunostaining images for sham and LPS-lesioned mice treated with vehicle or VCE-003.2 at 20 mg/kg are shown at right (scale bar = 50 µm), including a specific inlet showing the morphological characteristics of GFAP-labeled cells (2x magnified).

**Figure 6:**
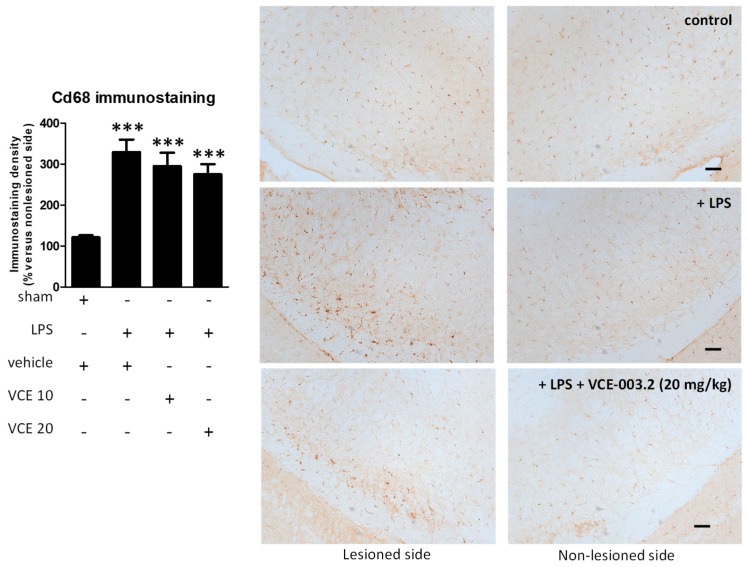
Intensity of the immunostaining for CD68 measured in a selected area of the substantia nigra pars compacta of control and LPS-lesioned mice orally treated for 28 days after lesion with vehicle (corn oil) or VCE-003.2 at the doses of 10 (VCE 10) or 20 (VCE 20) mg/kg. Immunoreactivity values are measured in the lesioned side over the non-lesioned side, and correspond to means ± SEM of more than 6 subjects per group. Data were assessed using the one-way analysis of variance followed by the Bonferroni test (*** *p* < 0.005 versus vehicle-treated control (sham) mice). Representative immunostaining images for sham and LPS-lesioned mice treated with vehicle or VCE-003.2 at 20 mg/kg are shown at right (scale bar = 200 µm).

These changes were associated with a partial reduction triggered by VCE-003.2, at both doses, in the intense astroglial reactivity provoked by LPS in the substantia nigra, measured by GFAP immunostaining (F(3,47) = 17.16, *p* < 0.0001; Figure 5). In addition, treatment with VCE-003.2 reversed the ameboid characteristics of GFAP-labeled cells (increased cell volume and reduced length of processes), when activated by the LPS lesion, towards a more quiescent phenotype having lower cell volume and longer processes (see inlets in Figure 5). A modest trend towards a reduction was also observed for microglial reactivity labeled with CD68 (F(3,46) = 16.60, *p* < 0.0001; Figure 6), although the post-hoc test did not show statistically significant differences for the two doses of VCE-003.2 compared to vehicle-treated LPS-lesioned mice (Figure 5 and Figure 6).

**Figure 7:**
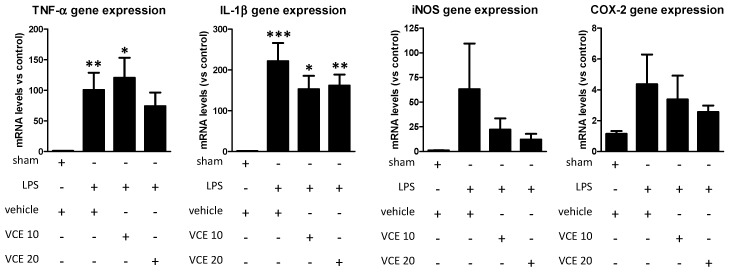
mRNA levels for TNF-α, IL-1β, iNOS, and COX-2 in the striatum of control and LPS-lesioned mice orally treated for 28 days after lesion with vehicle (corn oil) or VCE-003.2 at the doses of 10 (VCE 10) or 20 (VCE 20) mg/kg. Values were normalized versus control (sham) mice and correspond to means ± SEM of more than six subjects per group. Data were assessed using the one-way analysis of variance followed by the Bonferroni test (* *p* < 0.05, ** *p* < 0.01, *** *p* < 0.005 versus vehicle-treated control (sham) mice).

Lastly, the analysis by qPCR of proinflammatory mediators, such as tumor necrosis factor-α (TNF-α; F(3,48) = 5.149, *p* < 0.005) and interleukin-1β (IL-1β; F(3,46) = 10.59, *p* < 0.0001), in the striatum showed that the marked elevations provoked by the LPS lesion were, in general, attenuated by the treatment with VCE-003.2 with the greatest effects found with the dose of 20 mg/kg (except for IL-1β; Figure 7). Similar trends were found in relation with inducible nitric oxide synthase (iNOS; F(3,49) = 0.839, ns) and cyclooxygenase-2 (COX-2; F(3,51) = 1.031, ns) (Figure 7), although the differences did not reach statistical significance in most cases, partly due to the high variability in the lesioned groups, in particular in vehicle-treated LPS-lesioned mice.

## 3. Discussion

To determine whether glia-driven inflammation is a cause or a consequence of the degeneration of nigrostriatal neurons has remained an unsolved issue for years. At present, however, there is a general consensus assuming that glial activation may play an important pathogenic role in PD, contributing to the progressive degeneration of nigral dopaminergic neurons even in early phases of the disease [5,26]. This can be experimentally reproduced using LPS injection into the striatum [27], as well as in other experimental models of PD generated by classic mitochondrial neurotoxins [28], and it can also be seen in post-mortem PD brains at autopsy [4]. These observations prompted the investigation of anti-inflammatory agents as a potential disease-modifying therapy in PD. Thus, inhibitors of iNOS or COX-2, purinergic P2X receptor antagonists, pioglitazone, and other PPAR-γ activators, nonsteroidal anti-inflammatory drugs, the antibiotic minocycline, and immunosuppressants have been investigated at the preclinical and clinical levels in PD with variable success [4,5].

Cannabinoids have also been investigated, in particular those that activate the CB_2_ receptor [6,29], which experiences an intense up-regulatory response, predominantly in microglial cells and infiltrated macrophages recruited at Central Nervous System (CNS)-lesioned areas, in PD patients and experimental animal models [8,9]. The activation of this receptor in these experimental models has been associated with anti-inflammatory and neuroprotective effects [8,9,10]. More recent studies have proposed an alternative anti-inflammatory target for cannabinoids in PD, the PPAR-γ receptor, which has been found to play a relevant role in the control of inflammation in numerous pathological conditions [30,31]. In fact, classic activators of this receptor such as glitazones are currently under investigation in PD [14], whereas certain cannabinoids have been already investigated for their PPAR-γ-mediated anti-inflammatory activity in different models of central and peripheral inflammation [11]. With this idea in mind, we recently investigated a CBG derivative, VCE-003.2, which was previously found to activate PPAR-γ receptors, as anti-inflammatory and neuroprotectant in the classic inflammatory model of PD generated by intrastriatal application of LPS in mice [20]. LPS provoked an intense reactive microgliosis in the substantia nigra, in parallel to an elevated expression of proinflammatory markers (e.g., TNF-α, IL-1β, iNOS) in the striatum, resulting in the deterioration of TH-containing nigral neurons. The i.p. administration of VCE-003.2 reduced LPS-induced reactivity and toxicity of microglial cells with a partial recovery in the losses of TH-positive neurons in the substantia nigra [20]. We could confirm the involvement of PPAR-γ receptors in VCE-003.2 effects in LPS-lesioned mice, although our data demonstrate, using different experimental strategies (antagonism experiments, cell cultures, docking analysis), that the effects of VCE-003.2 are exerted through its binding to a regulatory site at the PPAR-γ receptor, which differs from the canonical binding site [20]. 

Given the therapeutic interest in VCE-003.2 against inflammation-driven neuronal deterioration, we attempted to further progress its validation as a potential neuroprotective agent in PD, by evaluating whether these beneficial effects are also found after oral administration, which is a more relevant route of administration for its clinical development compared to the i.p. route used in the previous study [20]. An oral lipid formulation of this compound was developed for this purpose. We used this formulation at two doses, 10 and 20 mg/kg, with the idea to reproduce the positive effects in the same experimental model of PD used in our previous experiment (LPS-lesioned mice) conducted with only one dose level (10 mg/kg) administered i.p. [20]. The dose of 10 mg/kg given orally was generally not active, with the only exception of GFAP labeling and reduction of IL-1β levels, and some small trends in behavioral tests, whereas oral administration of 20 mg/kg exhibited activity on many markers. VCE-003.2 at 20 mg/kg reduced LAMP-1 immunostaining, reflecting a positive effect on LPS-induced autophagy impairment, glial reactivity (mainly astrogliosis but also somewhat microgliosis), and the generation of pro-inflammatory markers. All these effects resulted in a partial recovery in the degeneration of TH-containing nigral neurons, as well as in a functional recovery reflected in the cylinder rearing test. Therefore, our data confirmed that oral VCE-003.2 was active, although a 2-fold higher dose of 20 mg/kg, or even higher, would be necessary to produce similar beneficial effects compared to the 10 mg/kg i.p. dose used in our previous study [20], and to progress towards the clinical development of this CBG derivative in PD. The differences between both studies may be due to the differences in bioavailability between oral and i.p. routes of administration, as well as to the fact that the present study was conducted in female mice in contrast to male mice used in the previous study [20]. The latter will require additional research.

## 4. Materials and Methods 

### 4.1. Synthesis and Formulation of VCE-003.2 

**Figure 8:**
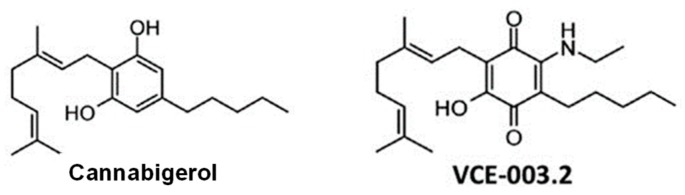
Chemical structures of cannabigerol (CBG) and VCE-003.2 (adapted from [19]).

The aminoquinone derivative of CBG (6-(3,7)-dimethyl-octa-2,6-dienyl)-5-hydroxy-3-pentyl-2-ethylamino-[1,4]benzoquinone), so-called VCE-003.2 was synthesized as described previously [19] (see chemical structures in Figure 8). Its activity as a PPAR-γ activator has also been previously characterized [19], as well as to its negligible affinity at the CB_1_ and CB_2_ receptors [20]. A lipid formulation of VCE-003.2 in corn oil was used for oral administration.

### 4.2. Animals and Surgical Lesions 

Female C57BL/6 mice (>7 month-old; 25–30 g weight) were housed in a room with controlled photoperiod (08:00–20:00) and temperature (22 ± 1 °C). They had free access to standard food and water. All experiments were conducted according to European guidelines (directive 2010/63/EU) and approved by the “Comité de Experimentación Animal” of our university (ref. PROEX 059/16). For in vivo experiments, mice (7–11 month-old) were anaesthetized (ketamine 40 mg/kg + xylazine 4 mg/kg, i.p.) and subjected to unilateral injections of S. Minnesota LPS (Sigma-Aldrich, Madrid, Spain) at two points of the right striatum following the procedure developed by Hunter et al. [27]. We used the following stereotaxic coordinates from bregma: +1.1 mm AP, −1.8 mm ML, and −3.5 mm DV, as well as −0.3 mm AP, −2.5 mm ML, and −3.2 mm DV (see details in [27]). At each intrastriatal coordinate, 5 μg of LPS in a volume of 1 μL of saline was injected slowly (0.5 µL/60 s) and the needle was left in place for 5 min before being slowly withdrawn. This avoids generating reflux and a rapid increase in intracranial pressure. Controls were sham-operated and injected with 1 μL of saline using the same coordinates. After the application of LPS or saline, mice were also subjected to pharmacological treatments as described in the following section. The lesions were generated using unilateral administration, allowing the contralateral structures to serve as controls for the different analyses.

### 4.3. Pharmacological Treatments and Sampling

LPS-lesioned mice were distributed into 3 groups, and administered orally with vehicle (corn oil) or 10 mg/kg or 20 mg/kg of VCE-003.2. The experiment included a fourth group consisting of sham-operated mice also treated orally with the same vehicle. The treatment was initiated approximately 16 h after the LPS lesion and was repeated daily for 28 days. Animals were subjected to behavioral analysis (rotarod, computer-aided actimeter) one week after the onset of treatment (post-lesion), and one day after the last administration (before autopsy). Immediately after the last behavioral testing, mice were killed by rapid and careful decapitation and their brains were rapidly removed and frozen in 2-methylbutane cooled in dry ice, then stored at −80 °C for subsequent immunohistochemical analysis in the substantia nigra and qPCR analysis in the striatum. In an additional experiment, the same treatment schedule was repeated using VCE-003.2, but only at the highest dose (20 mg/kg), with the purpose of exploring possible beneficial effects of this phytocannabinoid derivative at an additional behavioral measure, the cylinder rearing test, which may be more adequate for unilateral models of nigrostriatal damage.

### 4.4. Behavioral Analysis

Rotarod test: we used a LE8200 device (Panlab, Barcelona, Spain). After a period of acclimation and training (first session: 0 rpm for 10 s; second and third sessions: 4 rpm for 10 s), animals were tested with an acceleration from 4 to 40 rpm over a period of 300 s. Mice were tested for 3 consecutive trials and the mean of the 3 trials was calculated.

Computer-aided actimeter: motor activity was analyzed in a computer-aided actimeter (Actitrack, Panlab, Barcelona, Spain). This apparatus consisted of a 45 × 45 cm area, with infra-red beams all around, spaced 2.5 cm, coupled to a computerized control unit that analyzes the following parameters: (i) distance run in the actimeter (ambulation); (ii) time spent in inactivity; (iii) frequency of vertical activity (rearing); (iv) mean and maximal velocity developed during the running; and (v) time spent in fast (>5 cm/s) and slow (<5 cm/s) movements. Animals remained for a period of 10 min in the actimeter, but measurements were only recorded during the final 5 min (first 5 min was used only for animal acclimation).

Cylinder rearing test: given that the lesion with LPS was unilateral, this test attempts to quantify the degree of forepaw (ipsilateral, contralateral, or both) preference for wall contacts after placing the mouse in a methacrylate transparent cylinder of 15.5 cm in diameter and 12.7 cm in height [32]. Each score was made out of a 3 min trial with a minimum of 4 wall contacts.

### 4.5. Real Time qRT-PCR Analysis 

Brain coronal slices (around 500 µm thick) were made at levels containing the striatum, according to Palkovits and Brownstein Atlas [33]. Subsequently, such structure was dissected and frozen at −80 °C (using 2-methylbutane) up to be used for qRT-PCR analysis. Total RNA was isolated from the different samples using Trizol reagent (Sigma-Aldrich, Madrid, Spain). The total amount of RNA extracted was quantitated by spectrometry at 260 nm and its purity determined from the ratio between the absorbance values at 260 and 280 nm. After genomic DNA was removed (to eliminate DNA contamination), single stranded complementary DNA was synthesized from up to 1 μg of total RNA using the commercial kits RNeasy Mini Quantitect Reverse Transcription (Qiazen, Hilgen, Germany) and iScriptTM cDNA Synthesis Kit (Bio-Rad, Hercules, CA, USA). The reaction mixture was kept frozen at −20 °C until enzymatic amplification. Quantitative RT-PCR assays were performed using TaqMan Gene Expression Assays (Applied Biosystems, Foster City, CA, USA) to quantify mRNA levels for TNF-α (ref. Mm99999068_m1), IL-1β (ref. Mm00434228_m1), iNOS (ref. Mm01309902_m1), and COX-2 (ref. Mm00478372_m1), using GAPDH expression (reference Mm99999915_g1) as an endogenous control gene for normalization. The PCR assay was performed using the 7300 Fast Real-Time PCR System (Applied Biosystems, Foster City, CA, USA) and the threshold cycle (Ct) was calculated by the instrument’s software (7300 Fast System, Applied Biosystems, Foster City, CA, USA). Expression levels were calculated using the 2-ΔΔCt method.

### 4.6. Immunohistochemical Procedures 

Brains were sliced in coronal sections (containing the substantia nigra) in a cryostat (30 µm thick) and collected on antifreeze solution (glycerol/ethylene glycol/PBS; 2:3:5) and stored at −20 °C until used. Sections were mounted on gelatin-coated slides, and, once adhered, washed in 0.1 M potassium PBS (KPBS) at pH 7.4. Then endogenous peroxidase was blocked by 30 min incubation at room temperature in peroxidase blocking solution (Dako Cytomation, Glostrup, Denmark). After several washes with KPBS, sections were incubated overnight at room temperature with the following primary antibodies: (i) polyclonal rabbit anti-mouse TH antibody (Chemicon-Millipore, Temecula, CA, USA) used at 1/200; (ii) polyclonal rat anti-mouse CD68 antibody (AbD Serotec, Oxford, UK) used at 1/200; or (iii) polyclonal rabbit anti-mouse GFAP antibody (Dako Cytomation, Glostrup, Denmark) used at 1/200. In the case of LAMP-1 immunostaining, we used the hybridoma monoclonal rat antimouse LAMP-1 antibody 1D4B, which was deposited by Dr. J. Thomas in the Developmental Studies Hybridoma Bank (DSHB; Hybridoma Product 1D4B), created by the NICHD (NIH, Bethesda, MD, USA) and maintained at The University of Iowa, Department of Biology, Iowa City, IA, USA. In all cases, dilutions were performed in KPBS containing 2% bovine serum albumin and 0.1% Triton X-100 (Sigma Chem., Madrid, Spain). After incubation, sections were washed in KPBS, followed by incubation with the corresponding biotinylated secondary antibody (1/200) (Vector Laboratories, Burlingame, CA, USA) for 1 h at room temperature. The avidin–biotin complex (Vector Laboratories, Burlingame, CA, USA) and 3,3′-diaminobenzidine substrate–chromogen system (Dako Cytomation, Glostrup, Denmark) were used to obtain a visible reaction product. Negative control sections were obtained using the same protocol with omission of the primary antibody. A Leica DMRB microscope and a DFC300FX camera (Leica, Wetzlar, Germany) were used for the observation and photography of the slides, respectively. For quantification of the intensity of TH, LAMP-1, CD68, or GFAP immunostaining either in the substantia nigra (both ipsilateral and contralateral sides), we used the Image Processing and Analysis software ImageJ (U.S. NIH, Bethesda, MD, USA, http://imagej.nih.gov/ij/, 1997-2012) using 4 or 5 sections, separated approximately by 200 µm, and observed with 5–20x objectives depending on the method and the brain area under quantification. In all sections, the same area of the substantia nigra was analyzed. Analyses were always conducted by researchers who were blinded to all animal characteristics. Data were expressed as percentage of immunostaining intensity in the ipsilateral (lesioned) side over the contralateral (non-lesioned) side.

### 4.7. Data Analysis 

Data were assessed using one-way ANOVA followed by the Bonferroni multiple comparison test.

## 5. Conclusions

In summary, our data confirm the neuroprotective potential of an oral formulation of VCE-003.2 against inflammation-driven neuronal damage in an in vivo model of neuroinflammation reminiscent of PD. Our data also indicate the need for higher doses in the case of oral administration compared to our previous study with i.p. administration [20]. Although some additional confirmatory data are still required (e.g., evaluation in another model of PD), the present data support further development of such an oral product towards the clinic, to address the lack of disease-modifying therapies in PD.

## References

[1] Chen H., Jacobs E., Schwarzschild M.A., McCullough M.L., Calle E.E., Thun M.J., Ascherio A. (2005). Nonsteroidal antiinflammatory drug use and the risk for Parkinson’s disease. Ann. Neurol..

[2] Wahner A.D., Sinsheimer J.S., Bronstein J.M., Ritz B. (2007). Inflammatory cytokine gene polymorphisms and increased risk of Parkinson disease. Arch. Neurol..

[3] Lim S., Chun Y., Lee J.S., Lee S.J. (2016). Neuroinflammation in synucleinopathies. Brain Pathol..

[4] Hirsch E.C., Hunot S. (2009). Neuroinflammation in Parkinson’s disease: A target for neuroprotection?. Lancet Neurol..

[5] Tansey M.G., Goldberg M.S. (2010). Neuroinflammation in Parkinson’s disease: Its role in neuronal death and implications for therapeutic intervention. Neurobiol. Dis..

[6] Fernández-Ruiz J., Romero J., Ramos J.A. (2015). Endocannabinoids and neurodegenerative disorders: Parkinson’s disease, Huntington’s chorea, Alzheimer’s disease, and others. Handb. Exp. Pharmacol..

[7] Chiurchiù V., Leuti A., Maccarrone M. (2015). Cannabinoid signaling and neuroinflammatory diseases: A melting pot for the regulation of brain immune responses. J. Neuroimmune Pharmacol..

[8] Price D.A., Martinez A.A., Seillier A., Koek W., Acosta Y., Fernández E., Strong R., Lutz B., Marsicano G., Roberts J.L. (2009). WIN55,212-2, a cannabinoid receptor agonist, protects against nigrostriatal cell loss in the 1-methyl-4-phenyl-1,2,3,6-tetrahydropyridine mouse model of Parkinson’s disease. Eur. J. Neurosci..

[9] Gómez-Gálvez Y., Palomo-Garo C., Fernández-Ruiz J., García C. (2016). Potential of the cannabinoid CB2 receptor as a pharmacological target against inflammation in Parkinson’s disease. Prog. Neuropsychopharmacol. Biol. Psychiatry.

[10] García C., Palomo-Garo C., García-Arencibia M., Ramos J., Pertwee R., Fernández-Ruiz J. (2011). Symptom-relieving and neuroprotective effects of the phytocannabinoid Δ⁹-THCV in animal models of Parkinson’s disease. Br. J. Pharmacol..

[11] O’Sullivan S.E., Kendall D.A. (2010). Cannabinoid activation of peroxisome proliferator-activated receptors: Potential for modulation of inflammatory disease. Immunobiology.

[12] Fidaleo M., Fanelli F., Ceru M.P., Moreno S. (2014). Neuroprotective properties of peroxisome proliferator-activated receptor-α (PPARα) and its lipid ligands. Curr. Med. Chem..

[13] Agarwal S., Yadav A., Chaturvedi R.K. (2017). Peroxisome proliferator-activated receptors (PPARs) as therapeutic target in neurodegenerative disorders. Biochem. Biophys. Res. Commun..

[14] Carta A.R., Simuni T. (2015). Thiazolidinediones under preclinical and early clinical development for the treatment of Parkinson’s disease. Expert Opin. Investig. Drugs..

[15] Pistis M., Melis M. (2010). From surface to nuclear receptors: The endocannabinoid family extends its assets. Curr. Med. Chem..

[16] Fernández-Ruiz J., Sagredo O., Pazos M.R., García C., Pertwee R., Mechoulam R., Martínez-Orgado J. (2013). Cannabidiol for neurodegenerative disorders: Important new clinical applications for this phytocannabinoid?. Br. J. Clin. Pharmacol..

[17] Granja A.G., Carrillo-Salinas F., Pagani A., Gómez-Cañas M., Negri R., Navarrete C., Mecha M., Mestre L., Fiebich B.L., Cantarero I. (2012). A cannabigerol quinone alleviates neuroinflammation in a chronic model of multiple sclerosis. J. Neuroimmune Pharmacol..

[18] Carrillo-Salinas F.J., Navarrete C., Mecha M., Feliú A., Collado J.A., Cantarero I., Bellido M.L., Muñoz E., Guaza C. (2014). A cannabigerol derivative suppresses immune responses and protects mice from experimental autoimmune encephalomyelitis. PLoS ONE.

[19] Díaz-Alonso J., Paraíso-Luna J., Navarrete C., Del Río C., Cantarero I., Palomares B., Aguareles J., Fernández-Ruiz J., Bellido M.L., Pollastro F. (2016). VCE-003.2, a novel cannabigerol derivative, enhances neuronal progenitor cell survival and alleviates symptomatology in murine models of Huntington’s disease. Sci. Rep..

[20] García C., Gómez-Cañas M., Burgaz S., Palomares B., Gómez-Gálvez Y., Palomo-Garo C., Campo S., Ferrer-Hernandez J., Pavicic C., Navarrete C. (2018). Benefits of VCE-003.2, a cannabigerol quinone derivative, against inflammation-driven neuronal deterioration in experimental Parkinson’s disease: Possible involvement of different binding sites at the PPARγ receptor. J. Neuroinflammation.

[21] Hughes T.S., Giri P.K., de Vera I.M., Marciano D.P., Kuruvilla D.S., Shin Y., Blayo A.L., Kamenecka T.M., Burris T.P., Griffin P.R. (2014). An alternate binding site for PPARγ ligands. Nat. Commun..

[22] Plowey E.D., Chu C.T. (2011). Synaptic dysfunction in genetic models of Parkinson’s disease: A role for autophagy?. Neurobiol. Dis..

[23] Palomo-Garo C., Gómez-Gálvez Y., García C., Fernández-Ruiz J. (2016). Targeting the cannabinoid CB2 receptor to attenuate the progression of motor deficits in LRRK2-transgenic mice. Pharmacol. Res..

[24] Zheng H.F., Yang Y.P., Hu L.F., Wang M.X., Wang F., Cao L.D., Li D., Mao C.J., Xiong K.P., Wang J.D. (2013). Autophagic impairment contributes to systemic inflammation-induced dopaminergic neuron loss in the midbrain. PLoS ONE.

[25] Hockey L.N., Kilpatrick B.S., Eden E.R., Lin-Moshier Y., Brailoiu G.C., Brailoiu E., Futter C.E., Schapira A.H., Marchant J.S., Patel S. (2015). Dysregulation of lysosomal morphology by pathogenic LRRK2 is corrected by TPC2 inhibition. J. Cell Sci..

[26] Ouchi Y., Yoshikawa E., Sekine Y., Futatsubashi M., Kanno T., Ogusu T., Torizuka T. (2005). Microglial activation and dopamine terminal loss in early Parkinson’s disease. Ann. Neurol..

[27] Hunter R.L., Cheng B., Choi D.Y., Liu M., Liu S., Cass W.A., Bing G. (2009). Intrastriatal lipopolysaccharide injection induces parkinsonism in C57/B6 mice. J. Neurosci. Res..

[28] Gao X., Hu X., Qian L., Yang S., Zhang W., Zhang D., Wu X., Fraser A., Wilson B., Flood P.M. (2008). Formyl-methionylleucyl-phenylalanine-induced dopaminergic neurotoxicity via microglial activation: A mediator between peripheral infection and neurodegeneration?. Environ. Health Perspect..

[29] Fernández-Ruiz J., Moro M.A., Martínez-Orgado J. (2015). Cannabinoids in neurodegenerative disorders and stroke/brain trauma: From preclinical models to clinical applications. Neurotherapeutics.

[30] Laganà A.S., Vitale S.G., Nigro A., Sofo V., Salmeri F.M., Rossetti P., Rapisarda A., La Vignera S., Condorelli R., Rizzo G. (2016). Pleiotropic actions of peroxisome proliferator-activated receptors (PPARs) in dysregulated metabolic homeostasis, inflammation and cancer: Current evidence and future perspectives. Int. J. Mol. Sci..

[31] Croasdell A., Duffney P.F., Kim N., Lacy S.H., Sime P.J., Phipps R.P. (2015). PPARγ and the innate immune system mediate the resolution of inflammation. PPAR Res..

[32] Fleming S.M., Ekhator O.R., Ghisays V. (2013). Assessment of sensorimotor function in mouse models of Parkinson’s disease. J. Vis. Exp..

[33] Palkovits M., Brownstein J. (1988). Maps and Guide to Microdissection of the Rat Brain.

